# Mapping Quality Indicators in Primary Health Care: A Scoping Review of Contemporary Approaches and Persistent Measurement Gaps

**DOI:** 10.3390/healthcare14172880

**Published:** 2026-09-07

**Authors:** Christos Triantafyllou, Anastasia Ntikoudi, Anastasia Papachristou, Vion Psiakis, Valter R. Fonseca, Joao Breda

**Affiliations:** WHO Athens Quality of Care and Patient Safety Office, 10675 Athens, Greece; ntikoudia@who.int (A.N.); papachristoua@who.int (A.P.); psiakisv@who.int (V.P.); fonsecav@who.int (V.R.F.); rodriguesdasilvabred@who.int (J.B.)

**Keywords:** primary health care, quality indicators, quality measurement, performance assessment, Donabedian framework, patient-reported outcomes, health equity

## Abstract

**Background/Objectives**: Quality indicators are essential for evaluating and improving primary health care (PHC), but existing indicator sets differ considerably in their definitions, development methods, data requirements, and applicability across healthcare systems. This scoping review aimed to identify and synthesize quality indicators used to assess PHC services, describe the principal areas and methodological approaches represented, and identify persistent measurement gaps. **Methods**: A scoping review was conducted in accordance with the Preferred Reporting Items for Systematic Reviews and Meta-Analyses Extension for Scoping Reviews. PubMed/MEDLINE, Embase, and CINAHL were searched from inception to December 2025, with an updated search conducted on 27 August 2026. Targeted grey-literature searches of World Health Organization, United Nations Children’s Fund, and Organisation for Economic Co-operation and Development sources were also undertaken. Two reviewers independently screened the records and assessed potentially eligible full texts. Data were synthesized narratively and interpreted using the Donabedian structure–process–outcome framework, the seven World Health Organization dimensions of healthcare quality, and PHC-specific functions and content areas. **Results**: Twenty-three sources were included, comprising empirical studies, indicator-development and consensus studies, methodological reviews, national quality-improvement programmes, and international performance-measurement frameworks. Recurring areas included chronic disease management, preventive care, medication safety, access, continuity, service delivery, patient safety, efficiency, and healthcare utilization. Patient-reported outcomes and experiences, equity, social determinants of health, structural capacity, and broader outcomes meaningful to patients and populations were less consistently represented. Substantial heterogeneity in indicator definitions, reporting levels, data sources, and methodological approaches limited direct comparison across frameworks. **Conclusions**: PHC quality measurement remains extensive but fragmented. Future frameworks should balance structure, process, and outcome indicators, incorporate outcomes that matter to people, and combine standardized core measures with context-specific, feasible, valid, and actionable indicators.

## 1. Introduction

Primary health care (PHC) is the foundation of high-quality, equitable, efficient, and sustainable health systems and is central to achieving universal health coverage and the health-related sustainable development goals [1,2]. It represents the usual first point of contact between individuals and healthcare services and includes health promotion, disease prevention, early diagnosis, treatment, rehabilitation, chronic disease management, and coordination with other levels of care including long-term care [2]. Strong PHC systems have been associated with improved population health outcomes, higher quality and equity, greater responsiveness to the needs of patients and communities, and more efficient use of healthcare resources [3].

The delivery of high-quality PHC has become increasingly important due to population ageing, the increasing prevalence of multimorbidity and noncommunicable diseases, and the growing demand for continuous and person-centered care. Quality healthcare should be effective, safe, people-centered, timely, equitable, integrated, and efficient [4]. Nevertheless, the broad scope of PHC, the diversity of the populations it serves, and the differences among national healthcare systems make its quality difficult to assess consistently.

Quality indicators are important measurable elements used to determine whether healthcare structures, processes, and outcomes meet predefined evidence-based or consensus-derived standards. According to the Donabedian framework, structure indicators assess the resources and organizational conditions under which care is provided, process indicators examine the activities performed during care delivery, and outcome indicators measure the effects of care on patients and populations [5]. In PHC, these indicators may evaluate, among others, accessibility, continuity and coordination of care, preventive services, chronic disease management, medication safety, clinical effectiveness, efficiency, patient experience, equity, and health outcomes.

To provide meaningful information, quality indicators should be relevant, clearly defined, valid, reliable, feasible, measurable, and sensitive to changes in healthcare performance [4]. Their development should be supported by scientific evidence, clinical guidelines, expert consensus, and, where appropriate, the perspectives of patients and other stakeholders [4]. Well-designed indicators can support benchmarking, clinical audit, accreditation, accountability, policy evaluation, resource allocation, and quality-improvement interventions. However, inappropriate or poorly defined indicators may increase the burden of data collection without accurately reflecting the quality of care delivered [4].

International initiatives have attempted to establish more comprehensive and comparable approaches to PHC performance assessment. The World Health Organization (WHO) and UNICEF Primary Health Care Measurement Framework and Indicators provides a global framework covering health-system determinants, service-delivery processes, outputs, outcomes, impact, equity, quality, and resilience [6]. Nevertheless, existing indicator sets continue to differ considerably in their objectives, definitions, development methods, data sources, and applicability. Moreover, the implementation of quality indicators often depends on the availability of reliable electronic health records, administrative databases, standardized documentation, and appropriate governance mechanisms [6].

Recent regional initiatives have also adopted multidimensional approaches to quality measurement. The WHO Regional Office for Europe report Taking the Pulse of Quality of Care and Patient Safety in the WHO European Region applied 46 indicators covering governance and health-system functions, access, effectiveness, patient safety, people-centeredness, equity, efficiency, and population health outcomes [7]. Although the framework was not developed exclusively for PHC, it demonstrates the importance of linking service-level indicators with governance, health-system performance, and outcomes at the population level.

Previous evidence syntheses have approached PHC quality measurement from different perspectives. Ramalho et al. conducted an umbrella review of 33 systematic reviews and identified 727 quality indicators or groups of indicators. Most were classified as process measures, while chronic care and effectiveness were particularly prominent. That review provided an extensive catalogue of previously synthesized indicators but was not designed to examine in detail how contemporary national and international PHC measurement frameworks are developed, implemented, operationalized, or transferred across different health-system contexts [8]. More recently, de Melo Santos et al. systematically reviewed 14 peer-reviewed studies published between 2016 and 2023 addressing performance-measurement systems in PHC. Their findings emphasized substantial methodological heterogeneity, contextual adaptation, and limited evidence regarding longitudinal and post-implementation evaluation. However, their review focused primarily on integrated performance-measurement systems, excluded grey literature, and did not systematically map the breadth of PHC indicator frameworks against established structure–process–outcome and multidimensional quality frameworks [9].

Accordingly, an important gap remains between cataloguing individual indicators or describing performance-measurement systems and understanding how the broader body of PHC quality-measurement frameworks is distributed across established dimensions of quality. In particular, it remains unclear which measurement areas recur across different frameworks, how structure, process, and outcome measures are balanced, whether the seven WHO dimensions of healthcare quality are consistently represented, and which areas remain underdeveloped. The diversity of indicator definitions, data sources, development methods, and health-system contexts also raises questions regarding feasibility and transferability. A contemporary scoping review was therefore warranted to integrate evidence from both peer-reviewed studies and major grey-literature frameworks, incorporate recent global and regional measurement initiatives, and examine whether persistent gaps remain in domains that are increasingly emphasized in current PHC policy, including person-centred outcomes, equity, and broader population-relevant measures.

The present scoping review therefore aimed to identify and synthesize quality indicators and measurement frameworks used to assess PHC services, examine their methodological and implementation characteristics, and map their coverage across the Donabedian structure–process–outcome framework, the seven WHO dimensions of healthcare quality, and PHC-specific functions. The primary review question was: What quality indicators and measurement frameworks have been developed, selected, implemented, or evaluated for general PHC, and how do they map across established quality frameworks, methodological approaches, and areas of persistent measurement gap?

## 2. Materials and Methods

### 2.1. Review Design

A scoping review was conducted to identify, map, and synthesize the available evidence on quality indicators used in PHC. This methodological approach was selected because the literature includes heterogeneous indicator frameworks, study designs, development methods, data sources, and healthcare settings. The review focused on indicators developed, selected, implemented, or evaluated for the assessment of quality in general PHC services and examined their methodological characteristics, domains of application, and relevance to quality improvement. The review was conducted in accordance with the Preferred Reporting Items for Systematic Reviews and Meta-Analyses Extension for Scoping Reviews (PRISMA-ScR) [10,11].

### 2.2. Eligibility Criteria

Eligibility criteria were structured according to the Population–Concept–Context (PCC) framework. Because the primary unit of interest was the quality indicator, indicator set, or measurement framework rather than a predefined patient population, no specific participant-level population restriction was applied. Eligible sources could therefore involve individuals receiving PHC, healthcare professionals, practices, organizations, or health systems. The Concept comprised quality indicators, performance measures, indicator sets, monitoring systems, and structured assessment frameworks developed, selected, implemented, or evaluated for the assessment of PHC quality or performance. The Context comprised general primary health care, general practice, family medicine, community-based primary care, and other first-contact healthcare settings, without restrictions regarding geographical region or healthcare-system model.

Original research articles, methodological studies, observational evaluations, consensus and Delphi studies, feasibility and validation studies, and relevant national or international reports were eligible when they described the development, selection, implementation, application, or evaluation of PHC quality indicators. Methodologically detailed protocols were eligible when they described a structured process for developing or selecting PHC quality indicators. Protocols lacking sufficient methodological information or clearly identifiable indicator domains were excluded. Publications available in English were eligible for inclusion. A non-English full-text publication was retained when a sufficiently detailed English-language abstract was available to establish eligibility and extract the methodological and indicator-domain information required for the review. This applied to the study by Semlitsch et al. [12], for which the full text was published in German, but a detailed English-language abstract provided sufficient information for inclusion and data charting. The language restriction was a pragmatic eligibility decision intended to ensure consistent assessment and data charting across the included sources; nevertheless, it may have resulted in the omission of relevant national or regional frameworks published only in other languages.

Studies were excluded when the indicators focused exclusively on a single disease, such as diabetes mellitus, or on a specific age group or narrowly defined patient population, such as older adults. Studies addressing only hospital, inpatient, specialist, or emergency care were also excluded. However, comprehensive PHC frameworks that incorporated disease-specific or population-specific indicators as part of a broader multidimensional indicator set were retained.

### 2.3. Information Sources and Search Strategy

A structured literature search was conducted in PubMed/MEDLINE, Embase, and CINAHL. The initial searches covered each database from inception to December 2025 and were updated on 27 August 2026 before manuscript resubmission. PubMed/MEDLINE was searched via PubMed, Embase via Embase.com, and CINAHL via EBSCOhost. Database-specific controlled vocabulary and free-text terms related to healthcare quality indicators, performance measurement, and primary health care were combined using Boolean operators and adapted to the syntax of each database. Complete database-specific search strategies, including the exact search strings, controlled-vocabulary and free-text terms, field restrictions, Boolean operators, filters or limits, database platforms, and exact final search dates, are provided in Supplementary Table S2.

To complement the bibliographic database search, a targeted grey-literature search was conducted to identify relevant policy documents, institutional reports, and national or international PHC performance-measurement framework that might not be indexed in the bibliographic databases. The official websites of the World Health Organization (WHO), the United Nations Children’s Fund (UNICEF), and the Organisation for Economic Co-operation and Development (OECD) were searched using their internal search functions and combinations of the terms “primary health care”, “primary care”, “quality indicators”, “performance indicators”, “quality assessment”, and “measurement framework”. The grey-literature search was updated on 27 August 2026. Search results were screened against the same eligibility criteria applied to the bibliographic literature. The grey-literature search was intentionally restricted to these three organizations and to potentially eligible policy documents, institutional reports, and PHC measurement or performance frameworks. No predetermined numerical limit was applied to the number of records or documents screened within these website searches. For each organization, the exact search date, search procedure, and search terms are reported in Supplementary Table S2.

Source-specific retrieval counts for the individual bibliographic databases and organizational websites could not be reliably reconstructed retrospectively from the consolidated search records. Accordingly, only aggregate retrieval totals are reported in the PRISMA-ScR flow diagram.

### 2.4. Study Selection

All records identified through the database and grey-literature searches were compiled, and duplicate publications were removed before screening. Two reviewers independently assessed the titles and abstracts against the predefined eligibility criteria. Publications considered potentially relevant by either reviewer subsequently underwent independent full-text evaluation by the same two reviewers. Disagreements at either stage were resolved through discussion and consensus; when consensus could not be reached, a third reviewer was consulted. Full texts were sought through the corresponding database or publisher websites and, where necessary, through DOI- and title-based searches and available institutional access. Seventeen potentially relevant reports could not be retrieved despite these attempts and were therefore not assessed for eligibility. Sources were retained when they reported clearly identifiable PHC quality indicators or quality-assessment frameworks and provided sufficient information regarding their development, selection, validation, feasibility, implementation, application, or evaluation.

Publications that did not address quality measurement in general PHC settings, did not report identifiable indicators, or focused exclusively on an ineligible clinical setting or population were excluded. Reasons for exclusion at the full-text stage were documented. The study-selection process was summarized using a PRISMA-ScR flow diagram, presenting the numbers of records identified through bibliographic database and grey-literature searches, screened, assessed for eligibility, excluded, and included in the final review.

### 2.5. Data Charting and Classification

Data were charted independently by two reviewers using a standardized extraction form developed in accordance with the objectives of the review. Discrepancies between reviewers were resolved through discussion and consensus, with consultation of a third reviewer when agreement could not be reached. For each included source, the information recorded comprised the author or responsible organization, year of publication, geographical setting, study or framework focus, and the reported quality-indicator domains or categories. These characteristics are summarized in Supplementary Table S1. The extraction form was not formally pilot-tested; however, the reviewers discussed the charting fields and applied the same predefined fields consistently across all included sources.

Where the original source explicitly reported indicator domains or classifications, the original terminology was retained. When individual indicators were presented without broader domain headings, indicators were grouped into synthesized categories according to similarity in their underlying construct, intended measurement focus, and area of PHC quality, while preserving the terminology and meaning reported in the original source.

Because the included sources differed substantially in the level at which indicators were reported, ranging from individual measures to broad domains and composite frameworks, frequencies of individual indicators were not calculated. Indicators with apparently similar labels frequently differed in their definitions, numerators, denominators, data sources, and intended interpretation. A numerical aggregation would therefore have required subjective harmonization and could have overstated comparability across sources. This thematic grouping did not alter the content or definitions of the original indicators. Structure, process, and outcome classifications were retained where explicitly reported and were interpreted in accordance with the Donabedian framework [5].

### 2.6. Data Synthesis

The characteristics of the included studies and quality-indicator frameworks were synthesized descriptively and are presented in Supplementary Table S1. A narrative synthesis was undertaken to compare the methodological approaches used for indicator development and selection, the data sources required for their measurement, and their implementation across different PHC settings and healthcare systems.

The findings were interpreted using complementary analytical frameworks. First, reported indicators and domains were considered in relation to the Donabedian structure–process–outcome framework [5]. Second, the findings were examined in relation to the seven WHO dimensions of healthcare quality: effectiveness, safety, people-centredness, timeliness, equity, integration, and efficiency [4]. PHC-specific functions and content areas, including access and first-contact care, continuity, coordination, comprehensiveness, preventive care, chronic disease management, medication management, patient-reported outcomes, and community orientation, were considered separately to describe the substantive focus of the identified indicators. The Donabedian categories and WHO quality dimensions were applied using their published definitions as predefined operational frameworks. Where an included source explicitly classified its indicators according to structure, process, outcome, or a recognized quality dimension, the original classification was retained. Where such classifications were not explicitly reported, the frameworks were used to support interpretation of the narrative synthesis rather than to retrospectively impose a formal classification on each individual indicator.

For the source-level mapping, each included source was coded according to whether its reported indicators or domains addressed structure, process, and/or outcome within the Donabedian framework, the seven WHO dimensions of healthcare quality, and the predefined PHC-specific functions and content areas. Categories were not mutually exclusive, because individual sources could address multiple dimensions. Coding was performed at the source level rather than by quantitatively reclassifying every individual indicator. Where classifications were explicitly provided by the original source, these were retained; otherwise, the published definitions of the analytical frameworks were applied to the reported indicator content. Source-level classification was performed independently by the same two reviewers. The resulting classifications were subsequently compared, and disagreements were resolved through discussion and consensus.

Given the substantial heterogeneity in the level at which indicators and domains were reported across sources, the source-level framework coding was used to characterize patterns of coverage, whereas individual indicators were not quantitatively aggregated across sources. PHC-specific functions and content areas, including access and first-contact care, continuity, coordination, comprehensiveness, preventive care, chronic disease management, medication management, patient-reported outcomes, and community orientation, were coded separately. These categories were used to describe the substantive focus of the indicators and were not considered substitutes for the WHO quality dimensions.

Differences among frameworks were examined in relation to their objectives, methodological characteristics, data requirements, feasibility, and applicability across healthcare systems. Due to heterogeneity in indicator definitions, development approaches, and reported outcomes, quantitative synthesis and meta-analysis were not undertaken.

### 2.7. Consideration of Methodological Characteristics

A formal critical appraisal or risk-of-bias assessment was not undertaken, because the purpose of this scoping review was to map the range, characteristics, and applications of PHC quality indicators rather than to determine comparative effectiveness. Methodological characteristics relevant to indicator development and implementation, including the development approach, stakeholder involvement, data source, feasibility testing, validity or reliability assessment, and practical applicability, were considered when reported by the included sources. These characteristics were synthesized descriptively and were not used to exclude sources or assign an overall methodological-quality rating.

This scoping review was retrospectively registered on the Open Science Framework after completion of the literature search, study selection, data charting, and narrative synthesis (Registration DOI: 10.17605/OSF.IO/JSDU6). The registration documents the methods applied in the completed review and does not constitute prospective preregistration. The retrospective registration was undertaken to provide a permanent and transparent record of the review methods and analytical procedures applied, despite the fact that the review process had already been completed.

## 3. Results

**Figure 1:**
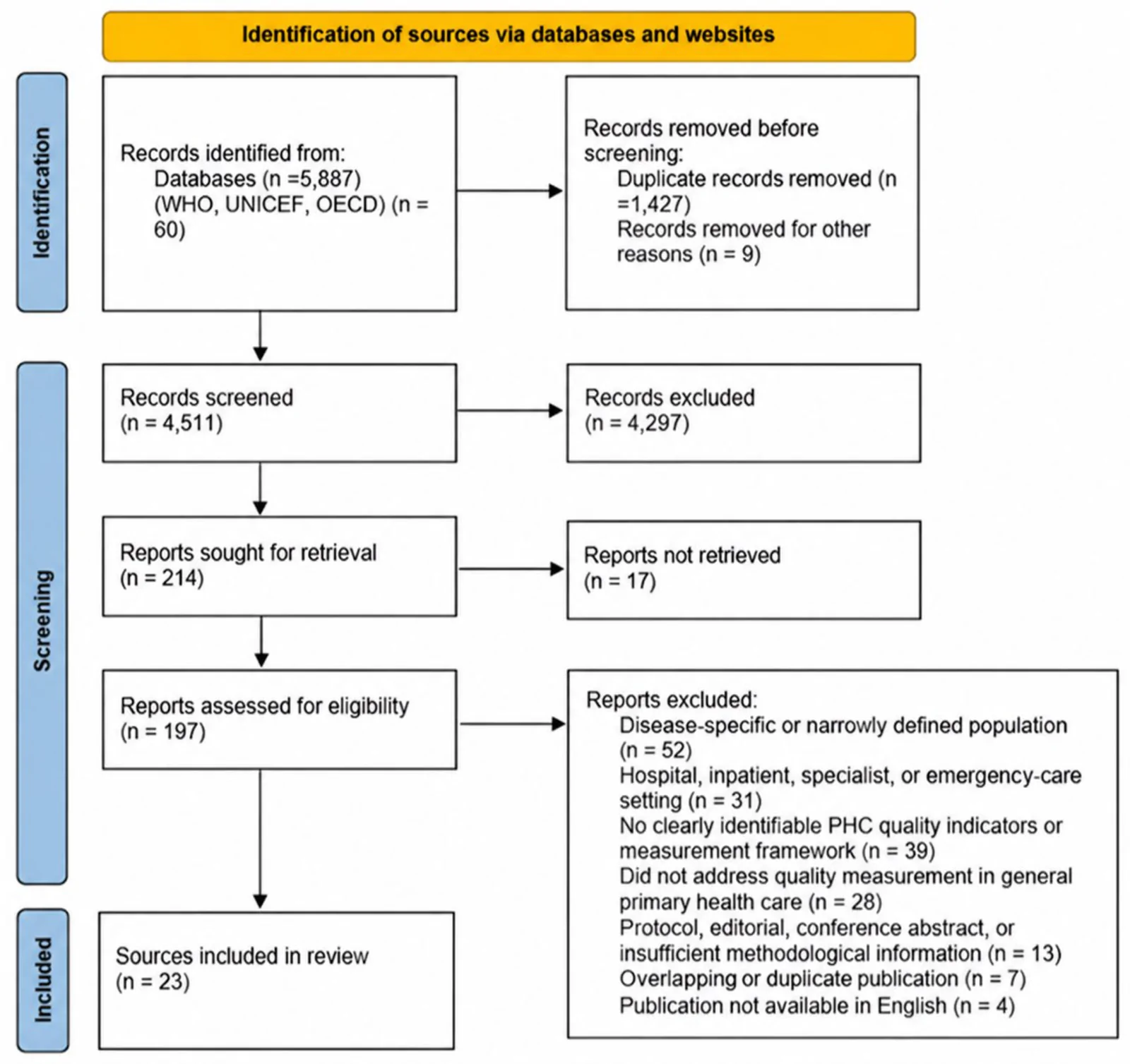
PRISMA-ScR flow diagram of the identification, screening, eligibility assessment, and inclusion of sources in the scoping review.

Twenty-three sources were included in the scoping review. The 23 included sources did not necessarily represent 23 independent indicator sets or frameworks, as some publications related to the same broader measurement programme or indicator-development initiative, while methodological reviews and appraisal studies examined multiple approaches (Figure 1). Consistent with the eligibility criteria, disease- or population-specific indicators were considered only when they formed part of a broader multidimensional PHC indicator set or measurement framework. Each publication or report was counted as one included source. Where multiple publications related to the same broader framework or indicator-development initiative, they were retained as distinct sources, and these relationships are described in the narrative synthesis where relevant. These comprised empirical and evaluative studies, indicator-development and consensus studies, methodological reviews, national quality-improvement initiatives, and national or international PHC performance-measurement frameworks. The included sources represented a range of geographical and health-system contexts, including country-specific initiatives from Europe, North America, the Asia-Pacific region, the Middle East, and Latin America, as well as multicountry and global frameworks. Their scope ranged from focused indicator sets addressing specific aspects of PHC quality to comprehensive frameworks covering multiple domains of primary care performance. The author or responsible organization, year of publication, geographical setting, study or framework focus, and reported quality-indicator domains of the included sources are presented in Supplementary Table S1. Table 1 summarizes the overall analytical pattern identified in the synthesis, while the source-level mapping of the 23 included sources across the Donabedian framework, WHO quality dimensions, and PHC-specific functions is presented in Supplementary Table S1.

### 3.1. Recurring and Underrepresented PHC Quality Areas

The descriptive synthesis was interpreted across three complementary perspectives: the Donabedian structure–process–outcome framework, the WHO dimensions of healthcare quality, and PHC-specific functions and content areas. Across the included sources, process-oriented aspects of care were recurrently represented, including chronic disease management, preventive care, medication management, access, continuity, and service delivery. Structural aspects were represented through organizational capacity, workforce, governance, and health-information infrastructure, whereas outcome-oriented measures included clinical outcomes, healthcare utilization, patient experience, and population-level outcomes. In relation to the WHO dimensions, effectiveness, safety, people-centredness, efficiency, equity, timeliness, and integration were represented to varying degrees across the identified frameworks. Patient-reported outcomes and experiences, equity, social determinants of health, and broader outcomes meaningful to patients and populations were less consistently incorporated. Within this qualitative synthesis, recurrence indicates representation across multiple heterogeneous sources rather than a formal numerical ranking. Chronic disease management, preventive care, medication management, access, continuity, and service delivery were represented across multiple study types and measurement frameworks. By contrast, explicit incorporation of PROMs, PREMs, equity-sensitive measures, social determinants of health, and broader patient- and population-relevant outcomes was confined to a more limited subset of the identified approaches.

The interpretive mapping therefore indicated a predominance of process-oriented measurement within the Donabedian framework, with structural and outcome-oriented measurement less consistently developed; across the WHO dimensions, all seven dimensions were represented, but people-centredness and equity were less systematically operationalized than more routinely measurable aspects of service delivery and clinical performance.

Chronic disease management was a recurring area among the included sources. Blozik et al. [13] developed 24 primary care quality indicators using health-insurance claims data in Switzerland, including indicators related to diabetes and cardiovascular disease. Huber et al. [14] examined associations between selected quality indicators and hospitalization risk, while Gribben et al. [15] developed population-based quality indicators in New Zealand with an emphasis on disease monitoring and feasibility in primary care.

Medication safety and prescribing were also represented in several included sources. Blozik et al. [13] included indicators addressing drug safety, medication use, and polypharmacy, particularly in older populations. Huber et al. [14] examined associations between prescribing-related indicators, polypharmacy, and hospitalization risk.

Access and continuity of care were addressed in several performance-measurement studies and frameworks. Al Rashidi et al. [16] evaluated 12 primary health care centers using six KPI components: accessibility, workload, outcomes, timeliness, satisfaction, and safety. Wessell et al. [17] reported benchmark data for 54 quality indicators derived from 87 practices, including measures related to patient follow-up and continuity of care. Access-related indicators were also included in the Pan-Canadian Primary Health Care Indicator Development Project [18].

Efficiency-related measures included healthcare utilization, potentially avoidable hospitalizations, and other aspects of resource use. Blozik et al. [13] and Huber et al. [14] included indicators related to hospitalization and healthcare utilization, while the Primary Health Care Performance Initiative incorporated measures concerning service delivery and system performance across low- and middle-income countries [19].

Patient safety was addressed in the framework developed by Frigola-Capell et al. [20] through a literature review and Delphi survey. The proposed indicators covered areas including workforce and organizational factors, treatment procedures, and patient-safety practices in primary care.

Patient perspectives were infrequently incorporated into the identified indicator frameworks. During pilot testing by Matsumura et al. [21], 65.4% of patients supported regular quality assessments and approximately 72% supported external quality reviews. These findings concerned patient acceptability of the quality-assessment process rather than the formal inclusion of patient-reported outcome measures or patient-reported experience measures. Overall, PROMs and PREMs were not systematically incorporated across the reviewed frameworks.

Equity was explicitly incorporated into some, but not all, of the included frameworks. Rezapour et al. [22] included “access and equity” as a formal dimension of the Iranian PHC quality-assessment framework, while the WHO and UNICEF Primary Health Care Measurement Framework and Indicators identified equity as a cross-cutting dimension [23]. Kringos et al. [24] considered equity during the development of the European Primary Care Monitor; however, equity was not retained as a separate dimension in the final indicator set and was instead reflected across areas including governance, economic conditions, and access. The Pan-Canadian framework also included indicators related to coverage and support for vulnerable populations, although equity was not presented as a distinct domain [18].

### 3.2. Indicator Development Approaches

Several included studies described structured approaches to the development or selection of PHC quality indicators. Matsumura et al. [21] developed indicators for primary care in Japan using expert focus groups, a modified Delphi appropriateness method, and subsequent pilot testing in six clinics. Hysong et al. [25] proposed a structured expert-based approach for selecting essential indicators of primary care quality. Semlitsch et al. [12] identified potential indicators through a systematic review and subsequently assessed their relevance and practicability for Austrian PHC settings through expert evaluation.

Marshall et al. [26] examined the selection of PHC quality indicators at the health-system level across OECD countries, distinguishing measures related to preventive care from those addressing diagnosis and treatment in primary care. Lau et al. [27] conducted a Delphi consensus study to validate indicators and measures of high-quality general practice within the Quality Equity and Systems Transformation in Primary Health Care (QUEST-PHC) framework in Australia. The final framework comprised 79 indicators and 128 corresponding measures, with consensus achieved regarding their relevance and feasibility for general practice.

### 3.3. Monitoring Tools and Systematic Frameworks

Kringos et al. [24] described the development of the European Primary Care Monitor, which was designed to generate comparable information on primary care systems across European countries. The framework included 99 indicators covering structural, process, and outcome aspects of primary care. Rezapour et al. [22] developed a national PHC quality-assessment framework for Iran through literature review, Delphi rounds, and expert consultation. The framework included access and equity, safety, efficiency, effectiveness, patient-centredness, governance, and appropriateness, with indicators further classified according to structure, process, and outcome. Rezapour et al. [28] subsequently conducted a state-of-the-art review of PHC quality-assessment frameworks and identified domains including health promotion and disease prevention, clinical management, maternal and reproductive health, access and service delivery, patient-centredness and community engagement; patient safety and medication management; workforce and collaborative practice; governance; and health-system capacity.

The Pan-Canadian Primary Health Care Indicator Development Project combined literature review, expert input, and stakeholder consultation to develop a broad set of PHC indicators for use at multiple levels [18]. Jurgutis et al. [29] examined primary care quality measurement in Lithuania in comparison with quality-improvement approaches used across the Baltic Sea Region and emphasized the involvement of patients, healthcare professionals, providers, and decision-makers in indicator selection. The Primary Health Care Performance Initiative (PHCPI) included 36 performance indicators and 56 diagnostic indicators and was developed to support performance improvement across 135 low- and middle-income countries [19]. The framework also documented limitations in data availability for several measures, particularly those relating to core PHC functions.

The WHO and UNICEF Primary Health Care Measurement Framework and Indicators provides a global approach to monitoring PHC capacities and performance across health-system structures and inputs, service-delivery processes and outputs, health outcomes, and impact, with quality, equity, and resilience incorporated as cross-cutting dimensions [23]. The framework is intended to support adaptation of indicators to national priorities, health-system contexts, and measurement capacities. Frigola-Capell et al. [20] developed an international patient-safety indicator framework for primary care through literature review and a Delphi survey, covering organizational, clinical, and patient-safety processes.

### 3.4. Implementation and Application of Quality Indicators

Blozik et al. [13] developed 24 evidence-based quality indicators using Swiss health-insurance claims data, covering general aspects and efficiency, drug safety, geriatric care, respiratory disease, diabetes mellitus, and cardiovascular disease. Blozik et al. [30] subsequently updated the SQIPRICA claims-based indicator set, adding measures related to laboratory testing, osteoarthritis, drug safety, and general aspects of care. Huber et al. [14] evaluated the association between selected primary-care quality indicators and hospitalization risk using population-based data. Wessell et al. [17] reported benchmark data for 54 indicators based on records from 87 primary care practices. Gribben et al. [15] developed and assessed the feasibility of population-based primary-care quality indicators in New Zealand.

### 3.5. Methodological Characteristics and Practical Applicability

The included sources used heterogeneous methodological approaches to develop and evaluate PHC quality indicators. Several indicator sets were developed through literature reviews, expert consultation, consensus procedures, or Delphi methods, while others were derived from administrative claims, electronic health records, or routinely collected clinical data. Feasibility was examined through pilot testing or implementation in clinical settings in a subset of the included sources. Evidence concerning validity, reliability, and practical applicability was reported inconsistently, and no common methodological appraisal framework was applied across all sources. Consequently, these characteristics were synthesized descriptively rather than used to rank the included indicators or frameworks.

### 3.6. Evaluative Studies and Performance Comparisons

Al Rashidi et al. [16] evaluated 12 primary health care centers using six KPI components: accessibility, workload, outcomes, timeliness, satisfaction, and safety. Perera et al. [31] developed and applied an evidence-based appraisal tool to assess the quality of a national set of primary-care performance indicators in New Zealand, identifying substantial variation in the methodological quality of the assessed indicators.

### 3.7. Policy-Level and National Initiatives

The National Program for Access and Quality Improvement in Primary Care (PMAQ) was established in Brazil to support improvements in access and quality in primary care [32]. The programme included 47 indicators organized across seven areas according to their function and use [32].

The OECD Health Care Quality Indicator (HCQI) project was initiated in 2001 to develop internationally comparable measures of healthcare quality [33]. Primary care was identified as one of the areas requiring further development within the indicator framework [33].

Laatikainen et al. [34] evaluated the feasibility of using a national electronic primary health care database in Kyrgyzstan to monitor population disease burden and the quality of care provided in PHC settings. The database enabled the extraction of indicators related to noncommunicable diseases and their management and demonstrated the potential use of routinely collected electronic PHC data for national performance monitoring.

## 4. Discussion

This scoping review mapped quality indicators and measurement frameworks applied across diverse PHC settings and identified substantial variation in their objectives, methodological approaches, indicator definitions, data sources, and levels of measurement. Some frameworks reported detailed individual indicators, whereas others used broader domains, composite measures, or health-system-level assessment frameworks. Overall, the findings illustrate the heterogeneity of current approaches to PHC quality measurement and the resulting challenges for direct comparison and transferability across healthcare systems.

The descriptive synthesis identified recurring attention to chronic disease management, preventive care, medication safety, access, continuity, service delivery, and healthcare utilization. This pattern is consistent with the umbrella review by Ramalho et al. [8], which found that PHC quality measurement predominantly relied on process indicators, while structure and outcome indicators were less extensively represented. The prominence of process-oriented measures may partly reflect their greater compatibility with routinely available clinical, administrative, insurance-claims, and electronic health-record data. In contrast, patient-reported outcomes and experiences, equity, social determinants of health, coordination, and broader patient- and population-relevant outcomes were incorporated less consistently.

Although several quality areas appeared repeatedly across the included sources, formal frequencies of individual indicators were not calculated. Indicators with similar titles frequently differed in their definitions, numerators, denominators, eligible populations, measurement periods, data sources, and intended interpretation. In addition, the included sources reported indicators at substantially different levels of aggregation. Combining these measures under common labels would therefore have required subjective harmonization and could have implied a level of comparability not supported by the original frameworks. The findings should consequently be interpreted as a thematic mapping of recurring measurement priorities and gaps rather than as a numerical ranking of individual indicators.

The underrepresentation of PROMs, PREMs, equity, and social determinants of health may reflect measurement constraints as much as differences in their perceived importance. Unlike many process indicators that can be generated from routinely collected administrative, claims, or clinical data, these domains often require additional surveys, standardized instruments, linkage across datasets, appropriate equity stratifiers, and dedicated data-governance arrangements. Routine PROM and PREM collection may also create additional financial and operational requirements related to survey administration, staff training, data management, analysis, and integration with existing information systems. These requirements may be particularly challenging in resource-constrained PHC systems. Equity and social-determinant measures also present challenges for cross-system standardization because relevant variables, definitions, data availability, and policy priorities differ across jurisdictions. Consequently, measurement systems may preferentially adopt indicators that are easier to obtain routinely, even when these provide a less complete representation of person-centredness, equity, and outcomes that matter to patients and populations [35,36].

For policymakers, indicator selection should balance standardization with feasibility and local relevance. A practical approach is to establish a parsimonious core set of indicators with common definitions and specifications that can support longitudinal monitoring and international comparison, while allowing additional measures to be selected according to national priorities, population needs, available data infrastructure, and service-delivery arrangements. Core indicators should therefore emphasize comparability and methodological consistency, whereas supplementary indicators can provide greater sensitivity to local context, equity concerns, and patient priorities. Where measurement capacity is limited, phased implementation can prioritize indicators that are both actionable and feasible before progressively incorporating more resource-intensive measures such as PROMs, PREMs, and linked equity-sensitive indicators. Periodic review of the indicator set is also important to ensure that measurement remains relevant and leads to quality-improvement action rather than becoming an administrative reporting exercise [37,38].

Some included evidence also suggests that selected quality indicators may be associated with clinically relevant outcomes. In the population-based cohort study by Huber et al. [14], performance on an HbA1c-related indicator was associated with a lower risk of hospitalization, whereas polypharmacy was associated with a higher risk. Given the observational design, these findings should be interpreted as associations rather than causal effects of quality-indicator implementation. More broadly, Renker-Darby et al. [39] reported that clinical indicators may help identify areas for improvement and support quality-improvement efforts when feedback is meaningful and healthcare teams have sufficient capacity to respond.

Persistent gaps were observed in structural measurement, patient-reported outcomes and experiences, equity, social determinants of health, and broader outcomes meaningful to patients and populations. This pattern is consistent with the broader PHC umbrella review by Ramalho et al. [8]. A disease-specific systematic review by Bam et al. [40] similarly identified a predominance of process measures in indicators for cardiovascular-disease prevention in primary care; this finding is considered corroborative rather than representative of PHC quality measurement as a whole.

Although process measures remain important for assessing whether recommended care is delivered, they provide only a partial indication of whether PHC structures and clinical activities translate into improvements in health, functioning, well-being, or patient experience. Greater incorporation of PROMs, PREMs, functional outcomes, quality-of-life measures, equity-sensitive indicators, and population-level outcomes may therefore support a more comprehensive and people-centred approach to PHC quality measurement. This is consistent with the emphasis on outcomes that matter to people and populations described by Fonseca et al. [41].

The findings are also consistent with the broader direction of WHO and UNICEF PHC measurement initiatives. The Operational Framework for Primary Health Care outlines strategic and operational levers for strengthening PHC systems [2], while the Primary Health Care Measurement Framework and Indicators provides a structured approach to monitoring inputs, service-delivery processes, outputs, outcomes, impact, quality, equity, and resilience [6]. Together, these frameworks illustrate the need for multidimensional measurement approaches that link service-level performance with wider health-system capacity and population outcomes.

This scoping review has several limitations. First, the review was restricted primarily to English-language evidence; one German-language publication was retained because a sufficiently detailed English-language abstract was available, and relevant frameworks available only in other languages may therefore have been missed. Second, the included sources showed substantial heterogeneity in their methodological approaches, indicator definitions, data sources, and levels of aggregation, which limited direct comparison across indicator sets and precluded meaningful numerical synthesis of individual indicators. Third, although grey-literature sources were searched, some national, regional, unpublished, or locally disseminated PHC quality-measurement frameworks may not have been identified. In addition, 17 reports identified during screening could not be retrieved for full-text assessment, and it is therefore possible that potentially eligible evidence was missed. Finally, the variability in reporting across included sources limited the extent to which methodological characteristics such as validity, reliability, feasibility, and implementation could be compared systematically.

## 5. Conclusions

This scoping review highlights the breadth and heterogeneity of international efforts to assess PHC quality through indicator-based approaches. Existing frameworks frequently emphasize clinical processes, chronic disease management, preventive care, medication safety, access, continuity, and service delivery. In contrast, structural capacity, patient experiences, patient-reported outcomes, equity, social determinants of health, and broader outcomes meaningful to people and populations remain less consistently represented.

On the basis of the identified gaps, three priorities emerge. First, PHC measurement systems should establish a concise core set of valid, feasible, and clearly specified indicators spanning structure, process, and outcome. Second, this core set should be progressively complemented by patient-reported outcomes and experiences, equity-sensitive measures, and broader population-relevant outcomes. Third, implementation should be adapted to local data capacity and resource availability, with indicators linked to feedback and quality-improvement mechanisms rather than collected solely for reporting purposes.

Future PHC quality-measurement frameworks should combine structure, process, and outcome indicators and align them with recognized dimensions of healthcare quality. Countries and organizations should prioritize a concise core set of clearly defined indicators that supports monitoring and comparison while allowing the inclusion of context-specific measures reflecting national priorities, population needs, and health-system characteristics. Indicator specifications should clearly define the target population, numerator, denominator, data source, reporting frequency, and relevant equity stratifiers.

Patients, communities, healthcare professionals, policymakers, and other relevant stakeholders should be involved throughout indicator development and selection. Greater use of patient-reported outcome measures, patient-reported experience measures, functional outcomes, quality-of-life measures, and population health outcomes is also needed to ensure that quality assessment captures the effects of care that matter most to people.

Before widespread implementation, indicators should be evaluated for validity, reliability, feasibility, data quality, interpretability, and burden of collection. Their use should be linked to feedback, accountability, and continuous quality-improvement activities rather than limited to administrative reporting. Further research should examine whether improvements in indicator performance are associated with meaningful patient and population outcomes and identify which measures can be adapted reliably across different PHC systems.

## Figures and Tables

**Table 1:** Interpretive overview of PHC quality measurement identified across the included sources.

Analytical Perspective	Areas Identified in the Review
Donabedian—Structure	Organizational capacity, workforce, governance, information systems and data infrastructure
Donabedian—Process	Chronic disease management, preventive care, prescribing and medication safety, access, continuity, coordination and service delivery
Donabedian—Outcome	Clinical outcomes, hospitalization and healthcare utilization, patient experience and population-level outcomes
WHO quality dimensions	Effectiveness, safety, people-centredness, timeliness, equity, integration and efficiency were represented to varying degrees
PHC-specific functions/content areas	Access and first-contact care, continuity, coordination, comprehensiveness, preventive care, chronic disease management and medication management
Less consistently represented areas	PROMs, PREMs, equity, social determinants of health, structural capacity and broader patient- and population-relevant outcomes

## Data Availability

No new primary data were generated. The data extracted, charted, and classified from the included sources that support the findings of this scoping review are provided in the article and Supplementary Materials. Additional review data are available from the corresponding author upon reasonable request.

## Supplementary Materials

The following supporting information can be downloaded at: https://www.mdpi.com/article/10.3390/healthcare14172880/s1. Table S1: Characteristics of the included sources, reported primary health care quality-indicator domains, and source-level analytical mapping; Table S2: Complete database and grey-literature search strategies; Supplementary File S1: PRISMA-ScR checklist. Reference [42] is cited in the supplementary materials.

## Abbreviations

The following abbreviations are used in this manuscript:

CINAHL	Cumulative Index to Nursing and Allied Health Literature
HbA1c	Glycated hemoglobin A1c
HCQI	Health Care Quality Indicator
HEALTH-IQ	Development and Implementation of a Framework for Quality Care
MEDLINE	Medical Literature Analysis and Retrieval System Online
NCDs	Noncommunicable diseases
OECD	Organisation for Economic Co-operation and Development
PC Monitor	Primary Care Monitor
PHC	Primary health care
PHCPI	Primary Health Care Performance Initiative
PMAQ	National Program for Access and Quality Improvement in Primary Care
PREMs	Patient-reported experience measures
PRISMA-ScR	Preferred Reporting Items for Systematic Reviews and Meta-Analyses Extension for Scoping Reviews
PROMs	Patient-reported outcome measures
QUEST-PHC	Quality, Equity and Systems Transformation in Primary Health Care
SQIPRICA	Swiss Quality Indicator for Primary Care
UNICEF	United Nations Children’s Fund
WHO	World Health Organization
