# Supplementary material for: Mapping Quality Indicators in Primary Health Care: A Scoping Review of Contemporary Approaches and Persistent Measurement Gaps
Source: Healthcare (Basel). 2026 Sep 7;14(17):2880. doi: 10.3390/healthcare14172880 (PMC13565370; doi:10.3390/healthcare14172880)
Supplement: Supplementary file 1 [file healthcare-14-02880-s001.zip › healthcare-4515573-supplementary.pdf]

Supplementary Table S1. Characteristics of the included sources, reported primary health care quality-indicator domains, and source-level analytical mapping.;

Study/framework	Geographical setting and study/framework focus	Reported indicator domains/categories	Donabedian classification	WHO quality dimensions	PHC-specific functions/content areas
Jurgutis et al., 2011	Geographical setting: Lithuania<br>Study/framework focus: PHC quality indicators for a more sustainable healthcare system.	• Indicators for the structure<br>• indicators for the process<br>• indicators for the outcome-related with overuse of more expensive/higher levels of care.	Structure; Process; Outcome	Efficiency	NR
Kringos et al., 2010	Geographical setting: Europe<br>Study/framework focus: European Primary Care Monitor covering structure, process, and outcome indicators.	• Governance of the PC system (Structure)<br>• economic conditions of the PC system (Structure)<br>• PC workforce development (Structure)<br>• access to PC services (Process)<br>• continuity of care (Process)<br>• coordination of care (Process)<br>• comprehensiveness of PC (Process)<br>• quality of PC (Outcome)<br>• efficiency of PC (Outcome)<br>• equity in health (Outcome).	Structure; Process; Outcome	Equity; Integration; Efficiency	Access/first-contact care; Continuity; Coordination; Comprehensiveness
OECD (2015)	Geographical setting: OECD countries<br>Study/framework focus: Health at a Glance 2015 indicator set.	• Population health status and mortality<br>• Health risk factors<br>• Healthcare coverage, financial protection, and access<br>• Timeliness of care<br>• Quality and outcomes of care<br>• Health-system expenditure and resources	Structure; Process; Outcome	Effectiveness; Timeliness	Access/first-contact care
Matsumura et al., 2019	Geographical setting: Japan<br>Study/framework focus: development and pilot testing of quality indicators for primary care.	• Comprehensive care/standardized practice<br>• access<br>• communication<br>• coordination<br>• understanding of patient background.	Process	People-centredness; Integration	Access/first-contact care; Coordination; Comprehensiveness
Rezapour et al., 2019	Geographical setting: Iran<br>Study/framework focus: development of a national PHC quality framework.	• Access and equity<br>• safety<br>• efficiency<br>• effectiveness<br>• patient centeredness<br>• governance<br>• appropriateness.<br><br>Indicators were further presented as structure, process, and/or outcome measures.	Structure; Process; Outcome	Effectiveness; Safety; People-centredness; Equity; Efficiency	Access/first-contact care
Blozik et al., 2018	Geographical setting: Switzerland<br>Study/framework focus: evidence-based quality indicators using health-insurance claims data and a pragmatic consensus process.	• General aspects/efficiency<br>• drug safety<br>• geriatric care<br>• respiratory disease<br>• diabetes mellitus<br>• cardiovascular disease.	Process	Effectiveness; Safety; Efficiency	Chronic disease management; Medication management

Study/framework	Geographical setting and study/framework focus	Reported indicator domains/categories	Donabedian classification	WHO quality dimensions	PHC-specific functions/content areas
Blozik et al., 2022	Geographical setting: Switzerland<br>Study/framework focus: updated claims-based quality indicators developed by the SQIPRICA working group.	• General aspects/efficiency<br>• laboratory testing<br>• osteoarthritis<br>• drug safety.	Process	Effectiveness; Safety; Efficiency	Chronic disease management; Medication management
Marshall et al., 2006	Geographical setting: OECD countries<br>Study/framework focus: selection of indicators for health promotion, prevention, and primary care at the health-system level.	• Preventive care<br>• diagnosis and treatment: primary care.	Process	Effectiveness	Preventive care
Lau et al., 2025	Geographical setting: Australia<br>Study/framework focus: Delphi consensus studyfor selecting indicators of high-quality general practice within QUEST-PHC.	• Person centred care and patient-team relationship<br>• evidence-based comprehensive care: preventive health care<br>• evidence-based comprehensive care: chronic care<br>• acute care: prescribing safety<br>• professionally accountable multidisciplinary team-based continuing care coordinated and integrated with other services<br>• clinical governance<br>• staff training<br>• data-enabled practice quality improvement<br>• education, training and research to support quality and sustainability<br>• accountable to the community<br>• accountable to society.	Structure; Process	Effectiveness; Safety; People-centredness; Integration	Continuity; Coordination; Comprehensiveness; Preventive care; Chronic disease management; Medication management; Community orientation
Laatikainen et al., 2022	Geographical setting: Kyrgyzstan<br>Study/framework focus: use of a national electronic PHC database to monitor primary care performance.	• Risk factors<br>• process indicators<br>• outcome indicators.	Process; Outcome	Effectiveness	Chronic disease management
Wessel et al., 2008	Geographical setting: Not specified<br>Study/framework focus: achievable benchmarks for PHC quality indicators in a practice-based research network.	• Diabetes mellitus<br>• heart disease and stroke<br>• cancer screening<br>• immunizations<br>• respiratory/infectious disease<br>• adult mental health and substance abuse<br>• nutrition and obesity<br>• inappropriate prescribing in the elderly.	Process	Effectiveness; Safety	Preventive care; Chronic disease management; Medication management

Study/framework	Geographical setting and study/framework focus	Reported indicator domains/categories	Donabedian classification	WHO quality dimensions	PHC-specific functions/content areas
Rezapour et al., 2022	Geographical setting: International scope<br>Study/framework focus: state-of-the-art review of PHC quality-assessment frameworks.	• Health promotion and disease prevention<br>• Clinical management of chronic and communicable conditions<br>• Maternal, child, and reproductive health<br>• Access, coverage, and service delivery<br>• Patient-centeredness and community engagement<br>• Patient safety and medication management<br>• Workforce and collaborative practice<br>• Governance, quality management, and health-system capacity	Structure; Process	Effectiveness; Safety; People-centredness; Integration	Access/first-contact care; Coordination; Comprehensiveness; Preventive care; Chronic disease management; Medication management; Community orientation
Pan-Canadian Primary Health Care Indicator Development Project, 2006	Geographical setting: Canada<br>Study/framework focus: Pan-Canadian Primary Health Care Indicator Development Project.	• Access, attachment, and timeliness of primary health care<br>• Population needs assessment, coverage, and support for vulnerable populations<br>• Health promotion, risk reduction, and self-management support<br>• Preventive, maternal, child, and screening services<br>• Chronic disease management and clinical outcomes<br>• Mental health and substance use care<br>• Medication safety and clinical quality improvement<br>• Patient-centeredness, participation, communication, and satisfaction<br>• Continuity, coordination, and integration of care<br>• Health information systems and digital communication<br>• Workforce capacity, professional development, and staff well-being<br>• Team-based and interdisciplinary care<br>• Organizational capacity and service availability<br>• Efficiency, duplication, and resource utilization<br>• Financing, remuneration, and operational expenditure	Structure; Process; Outcome	Effectiveness; Safety; People-centredness; Timeliness; Equity; Integration; Efficiency	Access/first-contact care; Continuity; Coordination; Comprehensiveness; Preventive care; Chronic disease management; Medication management; PROMs/PREMs; Community orientation
Hysong et al., 2022	Geographical setting: Not specified<br>Study/framework focus: structured expert approach to selecting essential indicators of primary care quality.	• Objective 1: Access<br>• Objective 2: Clinician-Patient Partnership<br>• Objective 3: Technical Quality.	Process	Effectiveness; People-centredness	Access/first-contact care

Study/framework	Geographical setting and study/framework focus	Reported indicator domains/categories	Donabedian classification	WHO quality dimensions	PHC-specific functions/content areas
National Program for Access and Quality Improvement in Primary Care (PMAQ)	Geographical setting: Brazil<br>Study/framework focus: National Program for Access and Quality Improvement in Primary Care (PMAQ).	• Women's health<br>• child health<br>• diabetes mellitus and systemic arterial hypertension control<br>• oral health<br>• general production<br>• tuberculosis and leprosy<br>• mental health.<br><br>Indicators were presented as performance and/or monitoring indicators.	Process; Outcome	Effectiveness	Comprehensiveness; Preventive care; Chronic disease management
Huber et al., 2020	Geographical setting: Switzerland<br>Study/framework focus: population-based cohort study examining evidence-based PHC quality indicators in relation to hospitalization risk.	• General aspects<br>• efficiency<br>• drug safety<br>• care for the elderly<br>• disease-specific indicators.	Process; Outcome	Effectiveness; Safety; Efficiency	Chronic disease management; Medication management
Perera et al., 2007	Geographical setting: Not specified<br>Study/framework focus: evidence-based tool for assessing performance indicators in primary health care.	• Phase 1 clinical indicators<br>• potential "second wave" clinical indicators.	Process	Effectiveness	NR
Al Rashidi et al., 2020	Geographical setting: Oman<br>Study/framework focus: cross-sectional observational assessment of key performance indicators in PHC centres.	• Accessibility<br>• workload<br>• safety<br>• outcome<br>• timeliness<br>• satisfaction.	Process; Outcome	Effectiveness; Safety; People-centredness; Timeliness	Access/first-contact care; PROMs/PREMs
Gribben et al., 2002	Geographical setting: New Zealand<br>Study/framework focus: population-based quality indicators for primary care.	• Health promotion and smoking cessation<br>• Cancer screening and immunization<br>• Blood pressure and cardiovascular risk assessment<br>• Appropriate prescribing and medication safety<br>• Cardiovascular disease management and secondary prevention<br>• Asthma and respiratory disease management<br>• Diabetes monitoring, complication screening, and risk management	Process	Effectiveness; Safety	Preventive care; Chronic disease management; Medication management
Semlitsch et al., 2019	Geographical setting: Austria<br>Study/framework focus: quality indicators developed for PHC facilities.	• Access<br>• quality of care<br>• continuity of supply<br>• coordination of care<br>• security	Process	Safety; Integration	Access/first-contact care; Continuity; Coordination
Frigola-Capell et al., 2015	Geographical setting: International scope<br>Study/framework focus: development of an international framework for patient-safety indicators in primary care through a literature review and online Delphi survey.	• Leadership<br>• people management<br>• partnerships and resources<br>• clinical processes.	Structure; Process	Safety	NR

Study/framework	Geographical setting and study/framework focus	Reported indicator domains/categories	Donabedian classification	WHO quality dimensions	PHC-specific functions/content areas
Veillard et al., 2017	Geographical setting: Low- and middle-income countries<br>Study/framework focus: development of the Primary Health Care Performance Initiative conceptual framework and selection of indicators for PHC performance measurement and improvement.	• System<br>• inputs<br>• service delivery<br>• outputs<br>• outcomes.	Structure; Process; Outcome	NR	NR
World Health Organization and United Nations Children's Fund, 2022	Geographical setting: Global<br>Study/framework focus: global measurement framework and indicator set for monitoring PHC capacities and performance through a primary health care lens.	• Health-system structures and inputs<br>• service-delivery processes<br>• service-delivery outputs<br>• health outcomes<br>• impact<br>• quality<br>• equity<br>• resilience.	Structure; Process; Outcome	Equity	NR

Abbreviations: PC, primary care; PHC, primary health care.

Table S2. Complete database and grey-literature search strategies

Information source	Platform / interface	Final search date	Complete search strategy / procedure	Limits / notes
PubMed/MEDLINE	PubMed	27 August 2026	("Primary Health Care"[Mesh] OR "primary health care"[Title/Abstract] OR "primary care"[Title/Abstract] OR "general practice"[Title/Abstract] OR "family medicine"[Title/Abstract] OR "community-based primary care"[Title/Abstract]) AND ("Quality Indicators, Health Care"[Mesh] OR "quality indicator*"[Title/Abstract] OR "performance indicator*"[Title/Abstract] OR "quality measure*"[Title/Abstract] OR "performance measure*"[Title/Abstract] OR "quality assessment"[Title/Abstract] OR "performance measurement"[Title/Abstract] OR "measurement framework*"[Title/Abstract] OR "quality framework*"[Title/Abstract])	From inception. No date restriction. Eligibility criteria, including language eligibility, were applied during screening.
Embase	Embase.com	27 August 2026	('primary health care'/exp OR 'primary health care':ti,ab OR 'primary care':ti,ab OR 'general practice':ti,ab OR 'family medicine':ti,ab OR 'community based primary care':ti,ab) AND ('quality indicator'/exp OR 'quality indicator*':ti,ab OR 'performance indicator*':ti,ab OR 'quality measure*':ti,ab OR 'performance measure*':ti,ab OR 'quality assessment':ti,ab OR 'performance measurement':ti,ab OR 'measurement framework*':ti,ab OR 'quality framework*':ti,ab)	From inception. No date restriction. Eligibility criteria, including language eligibility, were applied during screening.

Information source	Platform / interface	Final search date	Complete search strategy / procedure	Limits / notes
CINAHL	EBSCOhost	27 August 2026	(MH "Primary Health Care+" OR TI ("primary health care" OR "primary care" OR "general practice" OR "family medicine" OR "community-based primary care") OR AB ("primary health care" OR "primary care" OR "general practice" OR "family medicine" OR "community-based primary care")) AND ((MH "Quality of Health Care+") OR TI ("quality indicator*" OR "performance indicator*" OR "quality measure*" OR "performance measure*" OR "quality assessment" OR "performance measurement" OR "measurement framework*" OR "quality framework*") OR AB ("quality indicator*" OR "performance indicator*" OR "quality measure*" OR "performance measure*" OR "quality assessment" OR "performance measurement" OR "measurement framework*" OR "quality framework*"))	From inception. No date restriction. Eligibility criteria, including language eligibility, were applied during screening.
World Health Organization (WHO)	WHO website internal search	27 August 2026	Internal website search using combinations of: "primary health care"; "primary care"; "quality indicators"; "performance indicators"; "quality assessment"; "measurement framework". Potentially eligible policy documents, institutional reports, and PHC measurement/performance frameworks were screened against the same eligibility criteria as the bibliographic literature.	Targeted grey-literature search. No predetermined numerical limit on documents screened.
United Nations Children's Fund (UNICEF)	UNICEF website internal search	27 August 2026	Internal website search using combinations of: "primary health care"; "primary care"; "quality indicators"; "performance indicators"; "quality assessment"; "measurement framework". Potentially eligible policy documents, institutional reports, and PHC measurement/performance frameworks were screened against the same eligibility criteria as the bibliographic literature.	Targeted grey-literature search. No predetermined numerical limit on documents screened.
Organisation for Economic Co-operation and Development (OECD)	OECD website internal search	27 August 2026	Internal website search using combinations of: "primary health care"; "primary care"; "quality indicators"; "performance indicators"; "quality assessment"; "measurement framework". Potentially eligible policy documents, institutional reports, and PHC measurement/performance frameworks were screened against the same eligibility criteria as the bibliographic literature.	Targeted grey-literature search. No predetermined numerical limit on documents screened.
